# Artificial Intelligence and Statistics: Just the Old Wine in New Wineskins?

**DOI:** 10.3389/fdgth.2022.833912

**Published:** 2022-01-26

**Authors:** Livia Faes, Dawn A. Sim, Maarten van Smeden, Ulrike Held, Patrick M. Bossuyt, Lucas M. Bachmann

**Affiliations:** ^1^Medical Retina Department, Moorfields Eye Hospital NHS Foundation Trust, London, United Kingdom; ^2^Medignition Inc., Research Consultants, Zurich, Switzerland; ^3^Health Data Research UK, London, United Kingdom; ^4^National Institute for Health Research (NIHR) Biomedical Research Centre for Ophthalmology, Moorfields Eye Hospital National Health Service (NHS) Foundation Trust and University College London (UCL) Institute of Ophthalmology, London, United Kingdom; ^5^Julius Center for Health Science and Primary Care, University Medical Center Utrecht, University of Utrecht, Utrecht, Netherlands; ^6^Department of Biostatistics, Epidemiology, Biostatistics and Prevention Institute, University of Zurich, Zurich, Switzerland; ^7^Department of Clinical Epidemiology, Biostatistics and Bioinformatics, Amsterdam Public Health Research Institute, Amsterdam University Medical Centers, Amsterdam, Netherlands

**Keywords:** artificial intelligence (AI), machine learning (ML), statistics, methodology, reporting guideline

## Introduction

**Figure 1:**
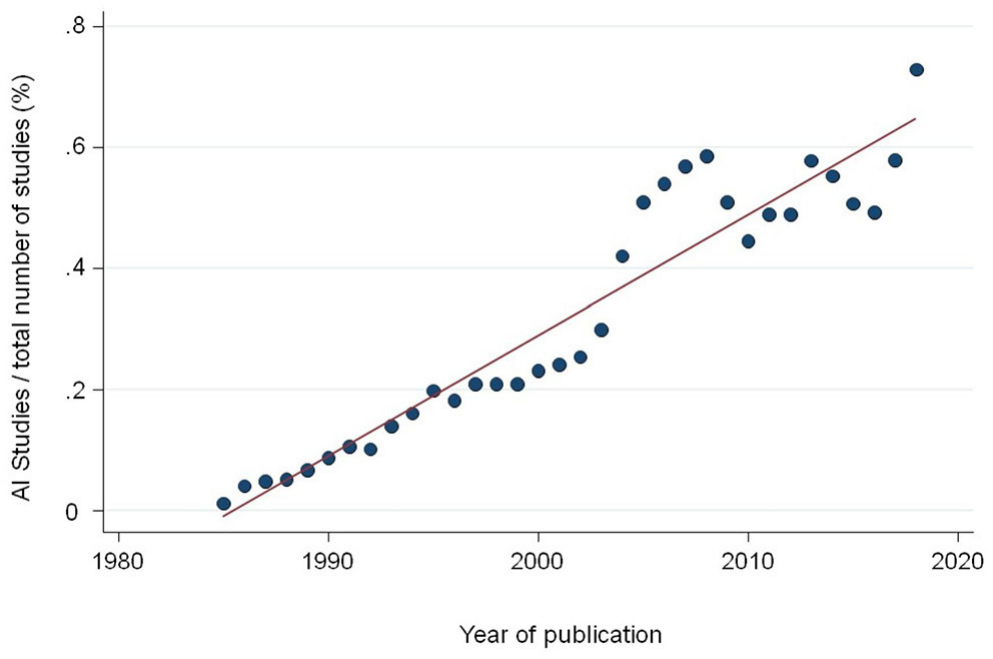
Proportion of studies indexed in Medline with the Medical Subject Heading (MeSH) term “Artificial Intelligence” divided by the total number of publications per year.

We are witnessing a tremendous increase in scientific studies in the medical literature using Artificial Intelligence (AI) and its branch Machine Learning (ML) methods in particular. A recent systematic review comparing the classification performance of healthcare professionals vs. AI retrieved over 20,000 records of study reports published since January 2012. In 2020 alone, over 7,000 new records were found in medical electronic databases (1). Simply by searching the Medline database using the Medical Subject Heading (MeSH) “Artificial Intelligence,” which was introduced in 1986, we find a continued increase of records over the last two decades (Figure 1). The total number of records currently indexed with the term adds up to 120,000 in Medline alone. Several issues beside the sheer number become apparent when reading through those papers.

## What Is the Difference Between AI and Statistics?

The general readership of medical journals, including clinicians, researchers, statisticians, and methodologists have experienced confusion with some of the terms they encounter in papers on AI. Table 1 shows a collection of terms found in the statistics world and its typical counterparts in the ML/AI field (2–4). A lack of consensus regarding terminology makes the comparability of studies and study results difficult, or even impossible. For example, in medical applications, diagnostic accuracy is usually reported using statistics as sensitivity, specificity, and area under the receiver operating characteristic curve. Studies using traditional statistical methods should report their results following the relevant reporting guidelines, such as STARD for diagnostic accuracy studies (5) and TRIPOD for prognostic models (6). In ML applications, models are commonly reported with other terms (i.e., recall and confusion matrix) but also different metrics (i.e., F1 score and dice coefficient). For example, reporting two by two tables of results for clinically relevant thresholds would achieve a higher comparability of studies by simple means.

**Table 1:** Dictionary of terms used in the statistical vs. machine learning/AI world.

**Statistical modeling**	**Machine learning/AI**
Estimating a model/Fitting	Learning
Prediction/Regression	Supervised learning
Latent variable modeling	Unsupervised learning
Case/Data point	Example/Instance
Sensitivity	Recall
Positive predictive value	Precision
Independent variable/Covariate	Feature
Dependent variable	Target
Response	Label
Parameters	Weights
Log likelihood	Loss
Structural equation model	Gaussian Bayesian network
Model for a categorical dependent variable	Classifier
Model for a continuous dependent variable	Regression
Model	Network, Graphs
Multinomial regression	Softmax
Prediction error	Error
Prediction of the sampling error	Variance
Average prediction error	Bias
Test set performance	Generalization
Contingency table	Confusion matrix
Criterion variable, reference test, gold standard	Ground truth
Overfitting	Overfitting
Measurement invariance	Transfer learning
Measurement error	Noise
Measurement error model (correction)	Noise aware machine learning
Measurement error model (estimation)	Inverse model
Deviance/Chi-square	Perplexity

Another conflict relates to the connection between AI and statistics. A growing number of researchers from various disciplines have expressed the view that many of the research questions are not too different in both disciplines (7). In fact, it may be argued that a large number of the differences in the analytical approach are only superficial and caused more by differences in terminology and scientific culture than from genuine dissimilarities (8). Differences may exist in terminology (not intentional) as they evolved in different scientific cultures with their legacy, nomenclature, notation, and philosophical perspectives (7, 9, 10).

## The Challenge of Developing a Common Scientific Language

Recently, several initiatives have been launched to advance the quality of reporting and the consistency of terminology in AI studies. It has been recognized that arriving at a consensus about a set of terms that could be used interchangeably between disciplines would reduce some of the unnecessary complexities which, for example, systematic reviewers might face when assessing different studies. Addressing these concerns, the Cochrane collaboration initiated the Cochrane Prognosis Methods Group (11), since “methodological development and refinement” was seen as crucial for future systematic reviews of prognostics studies. Also, guidelines for reporting development and validation of research using AI methodology (TRIPOD-AI and PROBAST-AI) are currently being developed by Collins et al. (12). Reporting guidelines for the early evaluation of AI systems (DECIDE-AI), performance evaluation (STARD-AI), and the evaluation in randomized controlled trials (CONSORT-AI) are also being developed (13, 14). The guidelines, using a principled approach including a consensus process among computer scientists, mathematicians, statisticians, healthcare professionals, epidemiologists, and clinicians, aim at improving completeness of reporting and shall assist researchers and policymakers when critically appraising the design, conduct, and analysis of ML based prediction model studies.

## The Chaos of Humans and Healthcare

At present, many of the algorithms frequently cited in the literature are hardly applicable in clinical practice (15). This is for two reasons: first, these AI innovations by themselves do not re-engineer the incentives that govern existing ways of working. A complex web of ingrained political and economic factors as well as the proximal influence of medical practice norms and commercial interests determine the way healthcare is delivered (16). Regulations and guidelines currently in use are not sufficient for AI methods to be reported in such detail that they can be reproduced and safely implemented in clinical practice for classification or prediction in new patients (17).

Perhaps with the exception of AI incorporated in Computer Aided Design (CAD) systems in radiology, simply adding AI applications to a fragmented system will not necessarily create sustainable change (18). However, by embedding AI applications into key drivers of a healthcare system, as was done within the National Health Service (NHS) in UK AI/automation could have real impact, if applied in a staged approach (19, 20).

Second, most healthcare organizations lack the data infrastructure required to collect the data that are needed for training algorithms so they can be (a) updated to the local population and/or the local practice patterns (a requirement prior to deployment that is rarely highlighted by current AI publications) and (b) for investigating the potential for biases, to guarantee that the algorithms perform consistently across patient cohorts, especially those who may not have been adequately represented in the training cohort (18). Additionally, the key regulators are still in consultation phase on how AI applications will be regulated, and what the level of validation needed is. There is also no assurance on how AI models will be monitored and audited in the event of adverse outcomes. Recent publications from the fields of sports medicine and oncology reflect these complexities (21, 22).

## The Ambitious Search for Suitable Areas of Application

Artificial intelligence and its branch ML have had their greatest successes in high signal/noise situations, e.g., visual and sound recognition, language translation, and playing games with concrete rules (23–28). What distinguishes these is rapid feedback while training, and availability of the correct answer. Things are different in the low signal/noise world and small datasets that typically prevail in diagnostic and descriptive prognostic research in medicine (29). A recent systematic review comparing AI with traditional statistical approaches found no advantages in terms of predictive accuracy between models developed with AI over logistic regression (9). Artificial intelligence can very well be applied in pattern recognition, to mimic or improve expert image interpretations. For estimating the probability of a positive biopsy given symptoms, signs, risk factors, and demographics, usefulness seems limited.

Currently, the most promising fields for AI applications are likely to be found in circumstances where the interpretation of estimated regression coefficients is not an issue, like in the context of triage based on image analysis. Efficient automated triage could reduce the burden of health services as it would identify a set of patients requiring timely care. Another area is AI application in less affluent countries where the lack of medical experts is a major impediment for an efficient delivery of healthcare (30, 31).

An essential difference between human and AI is that humans can learn efficiently even from small amounts of data. The neuronal processes involved are little known. A small child, for example, can recognize a leopard as a cat after looking at a few cat pictures. Machines generally need much more data to accomplish the same task. In addition, machines have no common sense. Although ML generally requires a large sample size, it is not clear how this can make accurate and unbiased predictions in erroneous data typically found in electronic medical records. Simply increasing the amount of data does not solve fundamental data quality problems (32).

On the other hand, AI algorithms can learn from huge amounts of data. While an AI model can be trained using a large amount of patient data from electronic patient files, a physician's ability to learn from experience is limited. Throughout his or her career, he or she will probably see only a fraction of the number of patients that can be offered to a ML model. Consequently substantial progress was made in clinical fields with large amounts of structured data such as pathology, radiology, and cardiovascular imaging. Results of classical comparative efficacy research and pragmatic studies have provided important insights for clinical practice by means of observational data. Recent attempts to use ML for this purpose have shown, however, that this is difficult because of the insufficient quality of the data sets. Moreover, the models need updating when new insights make this necessary. The claim to adapt these models due to regional differences in prescription practice is also hardly successful. The vision of automatically extracting the relevant variables from the electronic health record systems is promising but not yet a reality (33).

A promise of AI in health care is the avoidance of biases in arriving at diagnosis and in assigning medical treatment; a computer algorithm could objectively synthesize and interpret the data in the medical record. Integration of ML with clinical decision support tools, such as computerized alerts or diagnostic support, may offer physicians and others who provide health care targeted and timely information that can improve clinical decisions. The appropriateness of study data in terms of quality and representativeness is also of great importance in the world of ML. It is an unrealistic dream to believe that maximizing the amount of data automatically increases the quality of the data. The larger the proportion of errors in a data set, the more likely it is that the erroneous data and decisions will be represented in the model. The validity of data should always be distinguished from the quantity. While the former allows valid conclusions to be drawn, the latter only increases the precision of the potentially biased result (34, 35).

Artificial intelligence can be helpful in completing difficult tasks, such as the assessment of large amounts of imaging data. In order to develop its full potential, several steps need to be taken: First, joining the forces of various disciplines and stakeholders, and concerted efforts to reach a consensus about the terminology are needed. Second, once the various fields start using the same language, consolidation of the body of evidence will be feasible and specific requirements for sound research can be depicted. Third, results from AI research should be reported in way that directly inform clinical practice. Fourth, studies reporting the results of AI research should be methodologically comparable and accessible to meta-epidemiological assessment and economic evaluations.

## Discussion

It should be acknowledged that by the start of the twentieth century, medicine had moved from the empirical observation of individual cases to the scientific applications of today's research culture (36). Based on the fundamental findings of Pierre Simon Laplace, Ronald Fisher, Austin Bradford Hill, and other brilliant scientists, the body of knowledge that characterizes clinical research today was created (37). Fundamental work on ML goes back to the 1960s and was developed on the basis of mathematical and statistical principles to which traditional statistics also refers (38). It is worth remembering its roots.

The scientific community is called upon to make its contribution in knowledge development and cultivation. The recently launched initiatives will lay important foundations to make the findings from studies using these new forms of analysis more understandable, comparable, and critically assessable in the medium term (17). Ultimately, this goal is essential. Clinical research should never be an end in itself but should be at the service of improving medical care for the benefit of all.

## Author Contributions

LF and LB have drafted the manuscript. LF has created tables and figures. All authors have contributed to the conception to this opinion article, have reviewed, and edited the manuscript.

## Conflict of Interest

LB is employed by Medignition Inc., Research Consultants. The remaining authors declare that the research was conducted in the absence of any commercial or financial relationships that could be construed as a potential conflict of interest.

## Publisher's Note

All claims expressed in this article are solely those of the authors and do not necessarily represent those of their affiliated organizations, or those of the publisher, the editors and the reviewers. Any product that may be evaluated in this article, or claim that may be made by its manufacturer, is not guaranteed or endorsed by the publisher.
